# Histopathology-guided spatial transcriptomic analysis identifies an ECM-associated stromal pattern in lacrimal gland adenoid cystic carcinoma with complementary proteomic and functional analyses

**DOI:** 10.3389/fphar.2026.1949721

**Published:** 2026-10-09

**Authors:** Rong Luan, Difei Zuo, Yeqing Wang, Meixia Jiang, Xun Liu, Limin Zhu, Tingting Lin

**Affiliations:** 1 Tianjin Key Laboratory of Retinal Functions and Diseases, Tianjin Branch of National Clinical Research Center for Ocular Disease, Eye Institute and School of Optometry, Tianjin Medical University Eye Hospital, Tianjin, China; 2 Beijing Key Laboratory of Ophthalmology and Visual Sciences, Beijing Tongren Eye Center, Beijing Tongren Hospital, Capital Medical University, Beijing, China

**Keywords:** CD44, cell migration, extracellular matrix, lacrimal gland adenoid cystic carcinoma, spatial transcriptomics, tumor microenvironment, type I collagen

## Abstract

**Background:**

Lacrimal gland adenoid cystic carcinoma (LGACC) is a rare but locally aggressive malignancy characterized by perineural invasion, recurrence, and limited systemic treatment options. Although extracellular matrix (ECM) remodeling is increasingly recognized as an important regulator of tumor progression, its spatial organization and functional relevance in LGACC remain incompletely understood.

**Methods:**

A publicly available LGACC spatial transcriptomic tissue section was reanalyzed using histopathology-guided annotation to define a highly cellular Tumor Core and an adjacent stromal region, termed the Stroma Boundary. Regional differential expression and functional enrichment analyses were performed, together with module scoring, a sensitivity analysis within a shared transcriptomic cluster spanning both histopathologically defined regions, and CellChat-based ligand-receptor inference. The spatial findings were further contextualized using a public bulk RNA-sequencing cohort and an in-house quantitative proteomic cohort comparing conventional LGACC with LGACC showing high-grade transformation (LGACC-HGT). The effects of type I collagen were evaluated in SACC-LM cells using morphological assessment, CCK-8, wound-healing, Transwell migration, and Western blotting assays.

**Results:**

Within the analyzed spatial section, Stroma Boundary spots showed higher expression of ECM-related genes, including COL1A1, COL1A2, SPARC, and DCN, together with enrichment of collagen fibril organization and extracellular matrix organization. A secondary analysis restricted to a shared transcriptomic cluster spanning both histopathologically defined regions showed that ECM-associated differences remained detectable within a more similar transcriptomic background. CellChat analysis nominated collagen-associated interactions involving CD44 as candidate components of region-to-region communication. Bulk RNA-sequencing provided contextual support for the tumor-associated transcriptional program but incompletely captured the localized stromal pattern. Proteomic profiling demonstrated increased abundance of multiple collagen- and matrix-associated proteins in LGACC-HGT, accompanied by enrichment of ECM–receptor interaction and focal adhesion-related pathways. *In vitro*, type I collagen induced a more dispersed and spread cellular morphology and enhanced wound closure and Transwell migration without significantly altering short-term cell viability. Collagen exposure was also associated with increased CD44 protein expression. CD44 knockdown attenuated the COL1-induced increase in wound closure, suggesting a functional contribution of CD44 to this response.

**Conclusion:**

Histopathology-guided spatial analysis identified an ECM-enriched regional transcriptional pattern in the stromal compartment of the analyzed LGACC section. Complementary proteomic and functional findings suggest that collagen-associated remodeling may contribute to the migratory phenotype of ACC cells. CD44 represents a biologically plausible candidate associated with the collagen response, although its mechanistic role requires further validation.

## Introduction

Lacrimal gland adenoid cystic carcinoma (LGACC) is a rare yet highly aggressive epithelial malignancy of the ocular adnexa. Clinically, LGACC is characterized by an ominous disease course, with a marked propensity for perineural invasion, repeated local recurrence even after radical orbital exenteration, and delayed but frequently fatal distant metastasis (Emerick et al., 2022; Bradley and Bradley, 2013). Despite advances in multimodal treatment strategies, including aggressive surgical resection and radiotherapy, long-term outcomes remain dismal, particularly in patients with the solid histological subtype (Powell et al., 2023). This persistent clinical challenge is largely attributable to the paucity of actionable molecular targets and the intrinsic resistance of LGACC to conventional systemic chemotherapy. Drawing on findings from studies of adenoid cystic carcinoma (ACC) arising at other anatomical sites, emerging evidence suggests that the tumor microenvironment plays a pivotal role in regulating tumor progression and therapeutic resistance. This highly dynamic ecosystem comprises malignant cells, infiltrating immune cells, cancer-associated fibroblasts, and a continuously remodeled extracellular matrix (ECM) (Zhang et al., 2024; Peng et al., 2022). However, the precise spatial organization of these components within LGACC and the mechanisms through which they interact *in situ* to collectively confer the remarkable resilience of this malignancy remain largely unknown.

Previous molecular studies of LGACC have included conventional transcriptomic analyses and, more recently, spatial transcriptomic profiling (Jiang et al., 2021; Moeyersoms et al., 2023a). Although bulk RNA sequencing provides valuable molecular information, tissue homogenization removes the native spatial context of the tumor. By averaging transcriptional signals across heterogeneous cell populations, bulk profiling may obscure localized stromal programs and make the observed expression profile highly dependent on tissue composition. Consequently, biologically critical yet highly localized paracrine signals may be obscured by the overwhelming transcript abundance of the tumor core. These spatially confined structural and paracrine interactions are difficult to resolve using conventional bulk transcriptomic approaches, which lack information on the spatial organization of the tumor microenvironment. As a result, communication networks that are spatially segregated within the intact tissue become fundamentally masked. Resolving such profound intratumoral heterogeneity therefore requires spatially resolved approaches that integrate molecular profiling with the native histological context.

In this study, we hypothesized that spatially organized tumor-stromal interactions may contribute to the local biological heterogeneity of LGACC but are incompletely represented by conventional bulk sequencing approaches. To overcome this limitation, we reanalyzed a publicly available spatial transcriptomic (ST) section of LGACC to characterize histology-associated regional transcriptional patterns and integrated the resulting spatially resolved features with independent RNA-sequencing and quantitative proteomic dataset. This integrative analysis identified two morphology-defined spatial compartments within the analyzed tissue section: a highly cellular Tumor Core and an adjacent ECM-enriched Stroma Boundary. By integrating these within-section spatial observations with bulk transcriptomic, proteomic, and *in vitro* data, this study provides a hypothesis-generating framework for investigating ECM-associated tumor-stromal heterogeneity and matrix-responsive tumor cell behavior in LGACC.

## Materials and methods

### Data acquisition

Publicly available spatial transcriptomic and bulk RNA-sequencing datasets for LGACC were obtained from the Gene Expression Omnibus (GEO) database under accession numbers GSE228685 and GSE228914, respectively. An in-house quantitative proteomic dataset comparing LGACC and LGACC-HGT tissues was generated by our research group. The study involving human tissue specimens was approved by the Ethics Committee of Tianjin Medical University [approval no. 2019KY(L)-02] and was conducted in accordance with the Declaration of Helsinki. Written informed consent was obtained from all patients before tissue collection.

### Spatial transcriptomics data processing and histopathology-guided annotation

Raw spatial transcriptomic data generated using the 10 × Genomics Visium platform were loaded and processed with the Seurat R package. Spatial gene-expression counts were normalized using SCTransform to reduce technical variation while preserving biologically meaningful heterogeneity.

Histopathology-guided regional annotation was performed with reference to the matched high-resolution H&E-stained tissue section. The section was reviewed by two ophthalmic pathologists who were blinded to the spatial gene-expression results. Based on histomorphological features, spatial spots were manually annotated in Loupe Browser as either a highly cellular tumor region, termed the Tumor Core, or a fibrous stromal compartment surrounding or interposed between tumor nests, termed the Stroma Boundary. Spots for which the histomorphological assignment was not immediately clear were jointly reviewed and assigned by consensus. No formal quantitative interobserver concordance analysis was performed; discrepancies in regional assignment were resolved by consensus.

The term Stroma Boundary was used as an operational histopathological designation for regional comparison rather than as a distance-defined stromal subtype. Accordingly, no fixed geometric distance or predefined number of Visium spots from the tumor edge was used to define this region; instead, its spatial extent was determined according to the morphology of the fibrous stromal compartment on the matched H&E image. The spatial transcriptomic dataset comprised a single publicly available LGACC tissue section. Spatial spots were treated as observational units for exploratory within-section comparisons and were not considered independent biological replicates. Accordingly, spot-level statistical results were used to characterize regional transcriptional differences within the analyzed section and were not interpreted as population-level estimates across patients.

### Differential expression and functional enrichment analysis

To characterize the molecular differences between the two spatial niches, differential expression analysis was performed between the manually annotated Tumor Core and Stroma Boundary regions using the FindMarkers function in Seurat. Genes were considered significantly differentially expressed genes (DEGs) if they met the following criteria: an adjusted *P* value <0.05, an absolute average log_2_ fold change >0.25, and detectable expression in at least 10% of spatial spots (min.pct = 0.1). Representative region-associated DEGs were visualized using the ComplexHeatmap package. Tumor Core and Stroma Boundary spots were retained as separate histopathological groups and independently hierarchically clustered within each region using Euclidean distance and Ward’s method. Expression values were displayed as row-wise Z-scores. Subsequently, DEGs identified in each region were mapped to Entrez Gene identifiers using the org. Hs.e.g.,.db package. Gene Ontology biological process and Kyoto Encyclopedia of Genes and Genomes pathway enrichment analyses were then performed using the clusterProfiler R package. Enriched terms were considered statistically significant at an adjusted *P* value <0.05 and a *q* value <0.20, with multiple-testing correction performed using the Benjamini–Hochberg method.

### Spatially resolved region-to-region communication analysis

To investigate the paracrine and autocrine signaling networks coordinating the dual-niche tumor microenvironment, ligand-receptor-mediated communication was inferred using the CellChat R package. A CellChat object was constructed using the SCT-normalized spatial transcriptomic expression matrix and the histopathology-guided spatial annotations, with the human ligand-receptor interaction database (CellChatDB.human) selected as the reference. Overexpressed genes and ligand-receptor interactions were subsequently identified, and communication probabilities were estimated using the triMean method, which provides conservative and robust inference across groups of different sizes. Interactions involving spatial groups represented by fewer than 10 spots were excluded to reduce potentially unstable estimates. To characterize the directionality of signaling between the two spatial niches, we focused on the Collagen and MIF signaling pathways. Bidirectional signaling crosstalk between the Tumor Core and Stroma Boundary was visualized using gene-level chord diagrams (netVisual_chord_gene) and targeted bubble plots (netVisual_bubble).

### Sensitivity analysis within a shared transcriptomic cluster

As a secondary sensitivity analysis, we focused on a transcriptomically defined cluster that contained spatial spots from both the Tumor Core and Stroma Boundary (original transcriptomic Cluster 5). This cluster had been identified independently by unsupervised clustering of the spatial transcriptomic data and was not defined using the H&E-based regional annotations.

Differential expression analysis between the Stroma Boundary and Tumor Core within the shared transcriptomic cluster was performed using the same analytical framework as that applied in the primary region-based comparison. Representative ECM-associated genes, including COL1A1, COL1A2, SPARC, and DCN, were visualized using violin plots.

Regional transcriptional programs were further summarized using summary scores calculated with the AddModuleScore function in Seurat. The predefined ECM-associated gene set comprised COL1A1, COL1A2, COL3A1, COL6A1, COL6A2, SPARC, DCN, LUM, FN1, TNC, and FMOD. The predefined Tumor Core-associated gene set comprised MIF, MYC, CTNNB1, NCL, GABRP, and AZGP1. ECM-associated and Tumor Core-associated summary scores were calculated for each retained spatial spot and compared between Cluster 5 spots located in the Tumor Core and those located in the Stroma Boundary using the Wilcoxon rank-sum test. This cluster-restricted analysis was performed as a sensitivity analysis to determine whether the regional transcriptional patterns persisted within a shared graph-based transcriptional background. The shared transcriptomic cluster analysis was not used to define stromal identity or tumor proximity and was performed solely to assess whether the regional differences were entirely attributable to graph-cluster composition.

### Bulk transcriptomic contextualization in an independent cohort

An independent bulk RNA-sequencing cohort of LGACC (GSE228914), comprising two normal lacrimal gland samples and five LGACC samples, was analyzed to evaluate transcriptome-wide alterations. Raw count data were normalized and subjected to differential expression analysis using the DESeq2 R package. For volcano plot visualization, genes were considered significant based on an absolute fold change >1.5 and an unadjusted *P* value <0.05.

To assess whether spatially derived transcriptional features were also detectable at the bulk-tissue level, two custom gene signatures were constructed based on the spatial analysis. The Stroma Boundary signature comprised ECM-associated genes (COL1A1, COL1A2, COL6A1, COL6A2, COL6A3, COL9A1, SPARC, and FN1). The Tumor Core-associated custom signature, labeled “Spatial_Core_Survival” in Figure 1C, comprised MYB, MIF, CD44, CD74, and SDC4. This designation refers to a spatially derived Tumor Core-associated gene set. For the bulk RNA-seq cohort, genes were ranked according to the Wald statistics generated by DESeq2, and GSEA was performed using the clusterProfiler package to evaluate the enrichment patterns of these two custom spatial signatures.

**FIGURE 1:**
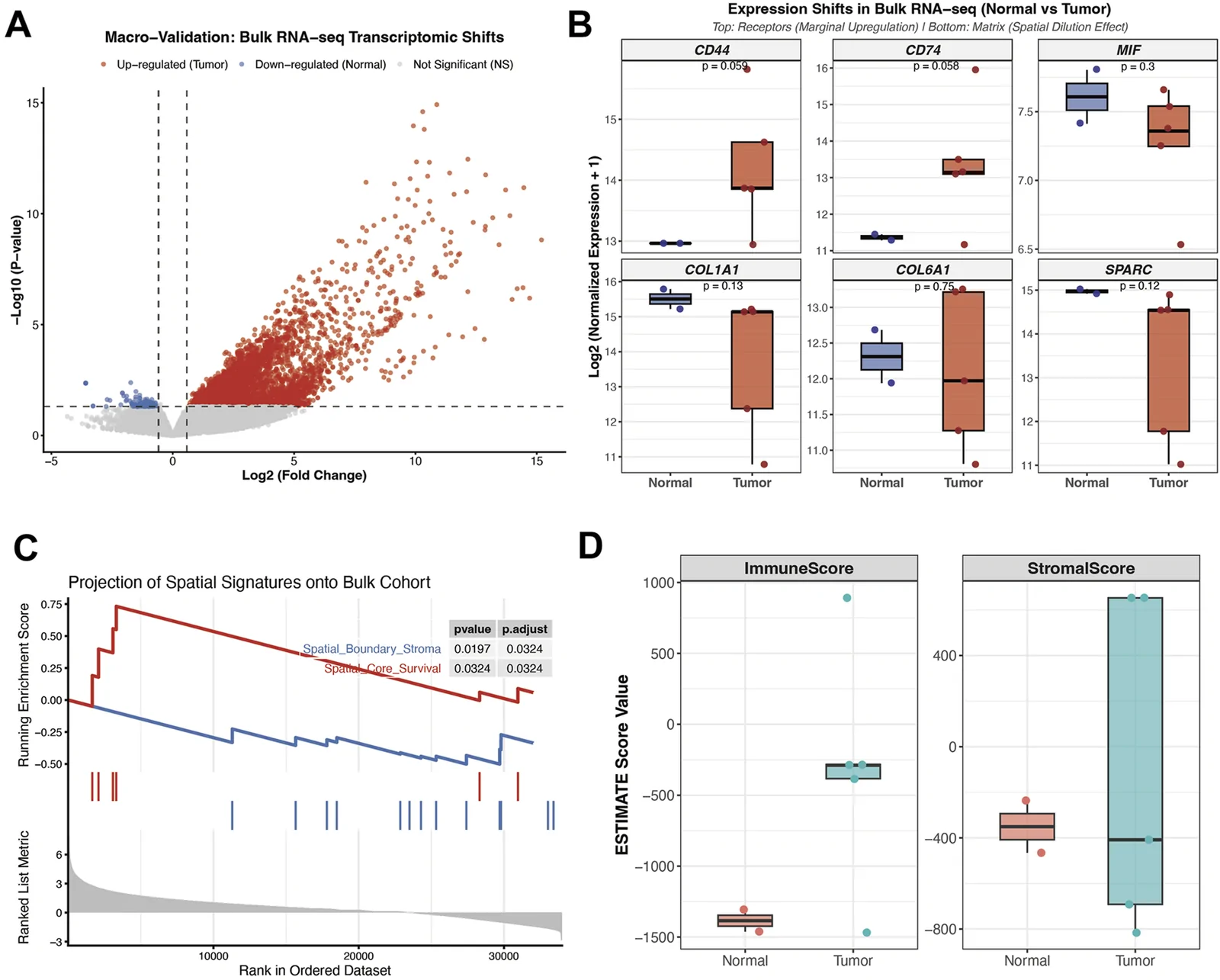
Bulk RNA-seq contextualizes spatially defined transcriptional programs in LGACC. **(A)** Bulk RNA-seq Transcriptomic Shifts. **(B)** Expression of representative receptor-related and ECM-associated genes in normal lacrimal gland and LGACC tissues. **(C)** Gene set enrichment analysis of spatially derived signatures in the bulk RNA-seq dataset. The Tumor Core-associated custom signature labeled “Spatial_Core_Survival” comprises MYB, MIF, CD44, CD74, and SDC4 and represents a spatially derived gene set. **(D)** ESTIMATE-derived ImmuneScore and StromalScore in normal lacrimal gland and LGACC samples.

### Tumor microenvironment analysis using ESTIMATE

To characterize stromal and immune components in the LGACC microenvironment, we applied the ESTIMATE (Estimation of STromal and Immune cells in MAlignant Tumours using Expression data) algorithm using the ‘estimate’ R package. Normalized gene expression matrices from the bulk RNA-seq data were used as input to calculate specific microenvironment scores for each sample. Specifically, the algorithm computes a StromalScore (reflecting stromal-associated gene-expression signals) and an ImmuneScore (reflecting immune-associated gene-expression signals). Statistical comparisons of these scores between different subgroups were performed using the Wilcoxon rank-sum test.

### Quantitative proteomics analysis

For the in-house LGACC proteomic cohort, protein abundance profiles were compared between tumors with and without high-grade transformation. Formalin-fixed, paraffin-embedded tissue specimens were collected from eight patients with LGACC who were treated at Tianjin Medical University Eye Hospital between December 2012 and January 2019. Based on histopathological evaluation, the patients were classified into a conventional LGACC group and an LGACC with high-grade transformation (LGACC-HGT) group, with four patients in each group. Detailed demographic and clinicopathological characteristics of the eight patients are provided in Supplementary Table S1.

Following deparaffinization, proteins were extracted from the tissue specimens and processed for downstream proteomic analysis. The extracted proteins were enzymatically digested into peptides, and peptide samples from different specimens were labeled with distinct isobaric tags for relative and absolute quantitation (iTRAQ). The labeled samples were fractionated and analyzed by liquid chromatography–tandem mass spectrometry (LC-MS/MS) using a Thermo Scientific Q Exactive mass spectrometer. Raw mass spectrometry data were processed using ProteinPilot software, version 5.0.2, and protein identification was performed against the *Homo sapiens* reference proteome in the UniProt database.

Based on the distribution of the proteomic data, proteins with a *P* value <0.05 and an absolute fold change >1.5 were defined as significantly differentially abundant proteins. To visualize the differential abundance of key structural extracellular matrix components and matrix-remodeling factors, customized lollipop plots and annotated volcano plots were generated using the ggplot2 and ggrepel R packages.

### Cell culture and extracellular matrix (ECM) coating

The human adenoid cystic carcinoma cell line SACC-LM was obtained from Shanghai Zhong Qiao Xin Zhou Biotechnology Co., Ltd. (catalog no. ZQ 0897) and was used at approximately passages 10–20. Mycoplasma-negative status was documented in the supplier-provided quality-control certificate. SACC-LM was cultured in DMEM/F12 medium supplemented with 10% fetal bovine serum and 1% penicillin-streptomycin in a humidified incubator at 37 °C with 5% CO_2_.

To recapitulate the collagen-rich microenvironment of the Stroma Boundary *in vitro*, culture plates were coated with type I collagen (COL1) using an air-drying method. The COL1 stock solution was diluted in 0.02 M acetic acid to achieve surface coating densities of 2 or 5 μg/cm^2^. The diluted collagen solution was added in a sufficient volume to completely cover the bottom of each well and was air-dried overnight in a biosafety cabinet. Before cell seeding, the collagen-coated plates were thoroughly washed three times with sterile phosphate-buffered saline (PBS) to remove residual acidic components.

To distinguish collagen-associated effects from those attributable to nonspecific enhancement of substrate adhesion, poly-L-lysine (PLL)-coated surfaces were additionally included as a non-collagen adhesive control. Cells cultured under uncoated, PLL-coated, and COL1-coated conditions were compared using wound-healings assays.

Cell viability was assessed using the Cell Counting Kit-8 (CCK-8) assay at 12 and 24 h after cell seeding and normalized to the corresponding 0-h baseline. To evaluate the effects of the collagen-rich matrix on the migratory properties of SACC-LM cells, wound-healing and Transwell migration assays were performed. For the Transwell migration assays, cells were seeded into the upper chambers of Transwell inserts with 8-μm pores. Three independent experiments were performed for each experimental condition.

#### siRNA-mediated CD44 knockdown

To assess the functional contribution of CD44 to the COL1-responsive phenotype, SACC-LM cells were transfected with CD44-targeting small interfering RNA (si-CD44) or negative-control siRNA (si-NC) (Shanghai Obio Technology [Group]Corp. , Ltd.). Knockdown efficiency was confirmed by Western blotting. Three experimental conditions were compared: si-NC-transfected cells cultured on uncoated surfaces, si-NC-transfected cells cultured on COL1-coated surfaces, and si-CD44-transfected cells cultured on COL1-coated surfaces. Cells were subjected to wound-healing assay to determine whether CD44 knockdown altered the COL1-responsive phenotype.

#### Western blotting

SACC-LM cells cultured on uncoated or type I collagen-coated plates were harvested and lysed for total protein extraction. Equal amounts of protein were separated by sodium dodecyl sulfate-polyacrylamide gel electrophoresis and transferred onto polyvinylidene fluoride membranes. After blocking, the membranes were incubated overnight at 4 °C with primary antibodies against CD44 (1: 1000, Proteintech, 15675-1-AP) and β-actin (1: 2000, CST, 4970). Band intensities were quantified using ImageJ software, and CD44 expression was normalized to β-actin. Six independent experiments were performed for each experimental condition.

#### Statistical analysis

All statistical analyses were performed using the R statistical computing environment (version 4.6.0) or GraphPad Prism. Continuous variables were compared between two groups using an unpaired two-tailed Student’s t-test or the Wilcoxon rank-sum test, as appropriate. Comparisons among multiple groups were performed using one-way analysis of variance (ANOVA) or the Kruskal–Wallis test, as appropriate. All statistical tests were two-sided, and a *P* value <0.05 was considered statistically significant.

## Results

### Histopathology-guided spatial transcriptomics identifies an ECM-associated Stroma Boundary pattern within the analyzed LGACC section

To examine regional transcriptional organization within an LGACC specimen, we analyzed a publicly available 10 × Genomics Visium spatial transcriptomic tissue section obtained from the GEO database (GSE228685). Guided by the matched H&E-stained tissue image, spatial spots were manually assigned to two regions: a highly cellular tumor region designated the Tumor Core and a stromal region immediately adjacent to the tumor nests, hereafter referred to as the Stroma Boundary (Figures 2A,B).

**FIGURE 2:**
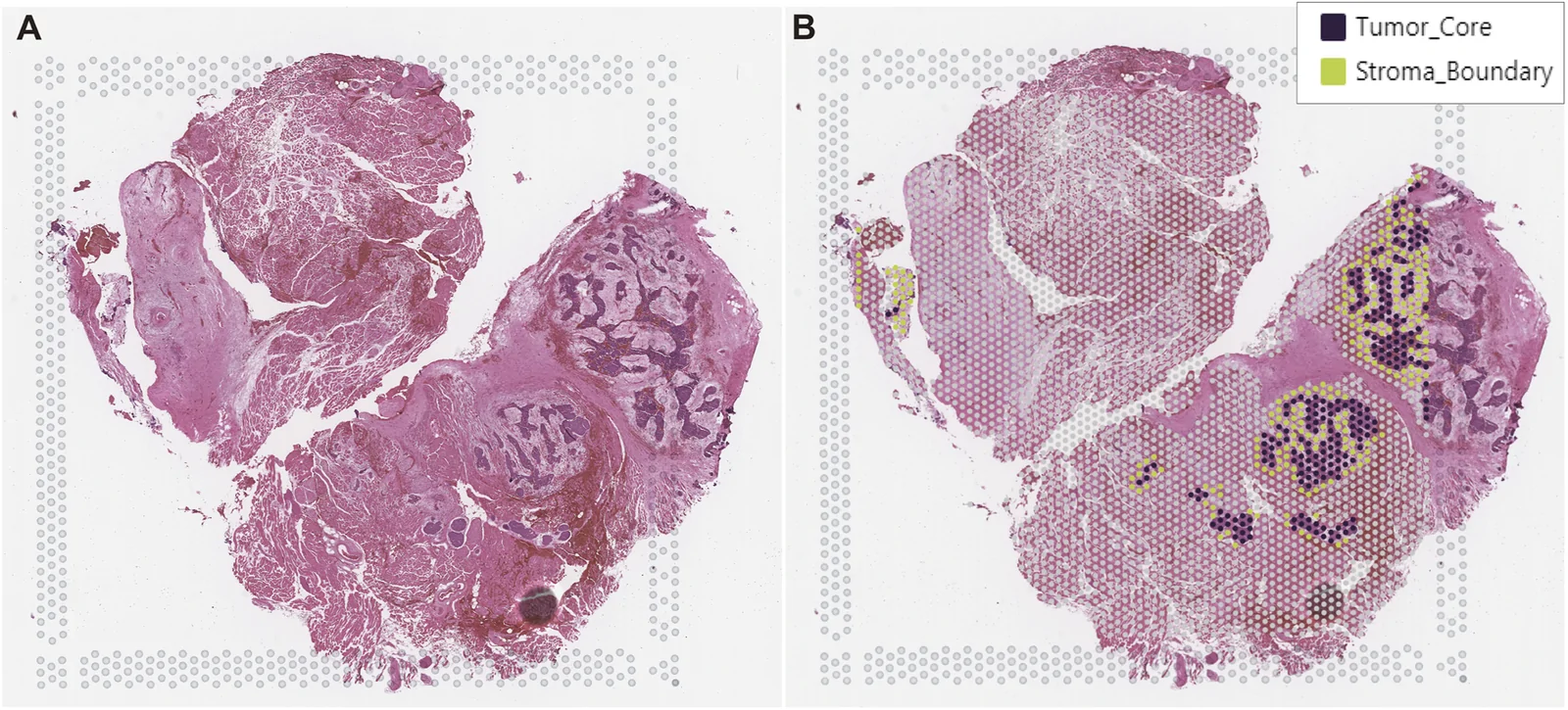
Histopathology-guided regional annotation of the analyzed LGACC spatial transcriptomic section. **(A)** Representative H&E-stained tissue section. **(B)** Histopathology-guided annotation of spatial spots into Tumor Core and Stroma Boundary regions.

Within the analyzed tissue section, Stroma Boundary spots showed higher expression of ECM-related genes, including COL1A1, COL1A2, SPARC, and DCN, than Tumor Core spots. In contrast, Tumor Core spots showed relatively higher expression of several genes associated with tumor-related transcriptional programs, including MYC and CTNNB1 (Figures 3A,B). Consistent with these findings, genes upregulated in the Stroma Boundary were predominantly enriched in biological processes related to collagen fibril organization, extracellular matrix organization, and extracellular structure organization (Figure 3C). By comparison, Gene Ontology enrichment of genes upregulated in the Tumor Core yielded only a limited set of significant terms, primarily related to RNA localization and related RNA-processing functions (Figure 3D). Each of these terms was supported by a small number of overlapping genes, and no canonical cancer-related pathway was significantly enriched. Accordingly, these Tumor Core enrichment results were interpreted descriptively rather than as evidence of a distinct cancer-specific signaling program. The restricted enrichment profile likely reflects the relatively limited number of Tumor Core-associated genes meeting the predefined differential-expression criteria. Collectively, these findings identify an ECM-associated regional transcriptional pattern within the histopathologically defined Stroma Boundary of the analyzed LGACC section. These spot-level comparisons reflect spatial heterogeneity within this individual tissue section and do not establish reproducibility of the pattern across LGACC patients.

**FIGURE 3:**
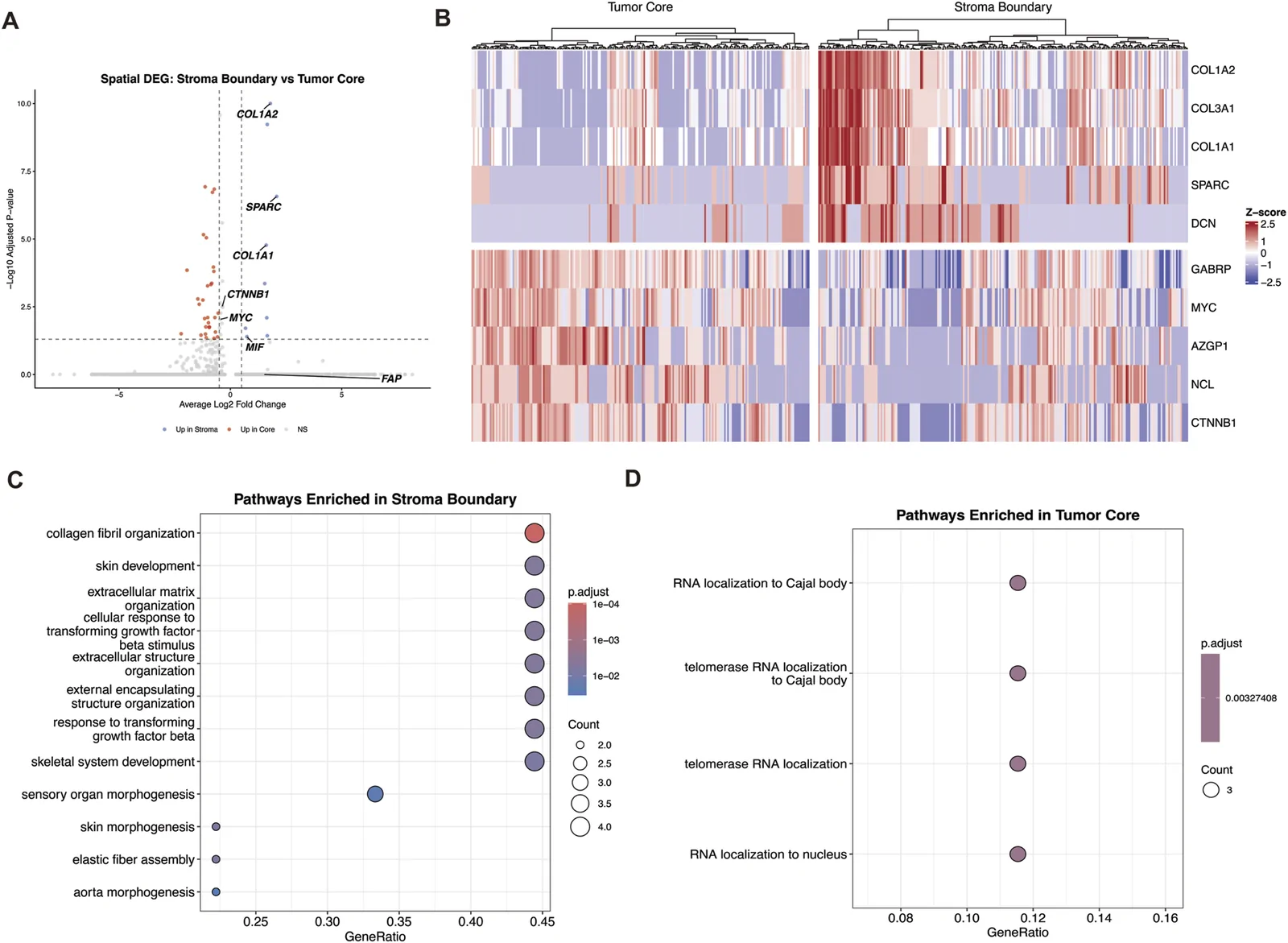
Histopathology-guided spatial analysis identifies an ECM-associated stromal regional pattern within the analyzed LGACC section. **(A)** Volcano plot of differentially expressed genes comparing Stroma Boundary with Tumor Core spots. Genes enriched in Stroma Boundary are shown in blue, whereas genes enriched in Tumor Core are shown in orange. **(B)** Heatmap showing representative differentially expressed genes across histopathology-guided Tumor Core and Stroma Boundary spots. **(C)** Gene Ontology biological process enrichment analysis of genes upregulated in Stroma Boundary. **(D)** Gene Ontology biological process enrichment analysis of genes upregulated in Tumor Core.

Because the original Loupe Browser dataset also contained graph-based clusters generated by unsupervised transcriptomic clustering, we further examined whether the regional differences described above could be attributable to differences in cluster composition. Spatial mapping showed that multiple graph-based clusters were distributed across the tumor-containing tissue region (Supplementary Figure S1A). Although the Tumor Core and Stroma Boundary exhibited partially distinct cluster compositions, Cluster 5 was the predominant cluster shared by both regions and was particularly prominent within the Tumor Core. In contrast, Clusters 2, 3, 6, and 7 made relatively greater contributions to the Stroma Boundary (Supplementary Figure S1B). We therefore restricted the subsequent analysis to spots assigned to Cluster 5, enabling comparison of the two histologically defined regions within their largest shared transcriptional background.

Among the graph-based clusters identified by unsupervised transcriptomic clustering, original transcriptomic Cluster 5 represented the predominant shared cluster spanning both the Tumor Core and Stroma Boundary. We next performed a secondary Cluster 5-restricted sensitivity analysis to determine whether the regional transcriptional differences were entirely explained by graph-cluster composition. Within the shared transcriptomic cluster of the analyzed section, COL1A1, COL1A2, SPARC, and DCN remained more highly expressed in Stroma Boundary spots than in Tumor Core spots (Supplementary Figure S1C). These findings indicate that the enrichment of collagen- and ECM-related transcripts in the Stroma Boundary was not solely attributable to the contribution of additional graph-based clusters within this region. Consistent with the gene-level results, the ECM-associated summary score remained significantly higher in Stroma Boundary spots than in Tumor Core spots within the shared transcriptomic cluster, whereas the predefined Tumor Core-associated summary score was higher in Tumor Core spots (Supplementary Figure S1D). Thus, the shared transcriptomic cluster analysis indicates that the observed Tumor Core-Stroma Boundary differences were not solely attributable to unequal graph-cluster composition. This analysis does not establish a distance-dependent stromal program.

Collectively, this sensitivity analysis showed that the ECM-associated regional pattern persisted within the predominant graph-based cluster shared by the two annotated regions in the analyzed section. Thus, the observed within-section difference was not solely attributable to unequal graph-cluster composition.

### Region-level ligand-receptor inference nominates candidate ECM-associated interactions

The histopathology-defined Tumor Core and Stroma Boundary showed different regional transcriptional patterns within the analyzed section. Based on this observation, we next used the histopathology-guided regional annotations to infer ligand-receptor relationships between the two regions using CellChat. Notably, this analysis was performed at the spatial-region level rather than at single-cell resolution. Accordingly, the inferred interactions should be interpreted as candidate region-to-region signaling relationships rather than definitive evidence of cell type-specific communication. Both the global interaction network and the bidirectional chord diagram indicated predicted signaling crosstalk between the two annotated regions, with Collagen- and MIF-related signaling pathways represented within the inferred communication network (Figures 4A,B).

**FIGURE 4:**
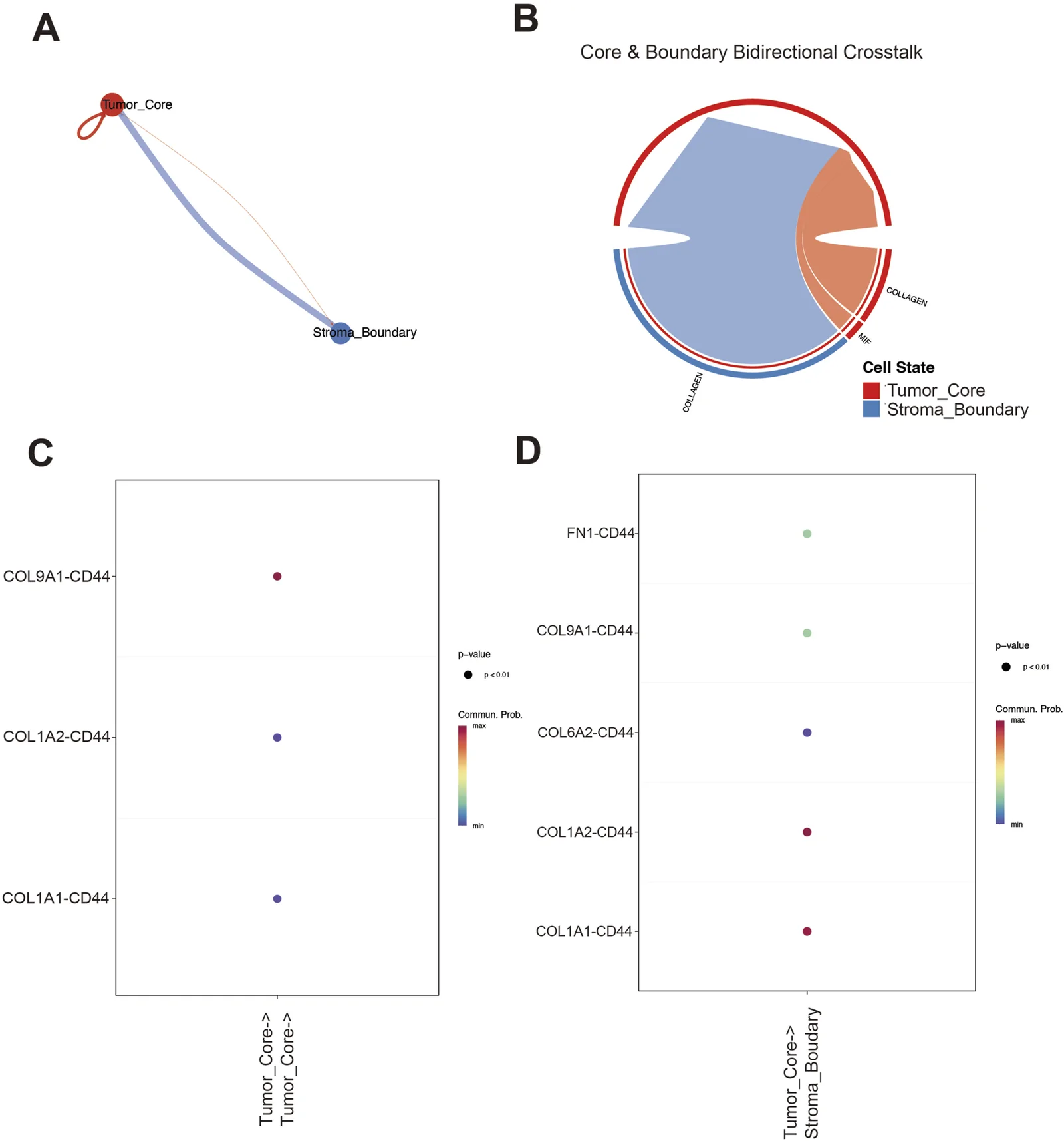
Predicted region-to-region signaling interactions between histopathologically defined Tumor Core and Stroma Boundary compartments. **(A)** Global CellChat-inferred interaction network between the histopathologically defined Tumor Core and Stroma Boundary regions. **(B)** Bidirectional chord diagram showing candidate signaling pathways between the two annotated regions. **(C)** Predicted collagen-CD44-related interactions within the tumor-core region. **(D)** Predicted ECM-associated ligand-receptor interactions from the Stroma Boundary to the Tumor Core, including collagen- and fibronectin-related ligands with CD44 as a candidate receiving receptor.

Further examination of ECM-associated candidate interactions involving CD44 suggested that the Stroma Boundary may transmit multiple collagen- and matrix-related ligand signals toward the Tumor Core, including COL1A1, COL1A2, COL6A2, COL9A1, and FN1. CD44 was identified as a candidate receptor for these signals within the Tumor Core (Figure 4D). In addition, collagen-CD44 interactions were also predicted within the Tumor Core itself (Figure 4C). These results nominate CD44 as a candidate receptor-associated component of the inferred ECM-related regional interaction network. However, because these observations were derived solely from transcriptome-based interaction inference, they do not directly demonstrate ligand binding, receptor activation, or functional downstream signal transduction.

To assess the spatial distribution of the selected signaling components, we mapped the expression of MIF and CD44 across the entire tissue section. Both genes exhibited heterogeneous spatial expression patterns and were detectable in tumor-associated regions, but neither was exclusively restricted to the Tumor Core or the Stroma Boundary (Figures 5A,B). MIF expression was relatively more prominent in tumor-associated regions, consistent with the trend toward its directional enrichment in the Tumor Core, whereas CD44 signals were detected across multiple tumor-associated regions. Thus, CD44 may represent a candidate signaling component shared between the Tumor Core and the Stroma Boundary.

**FIGURE 5:**
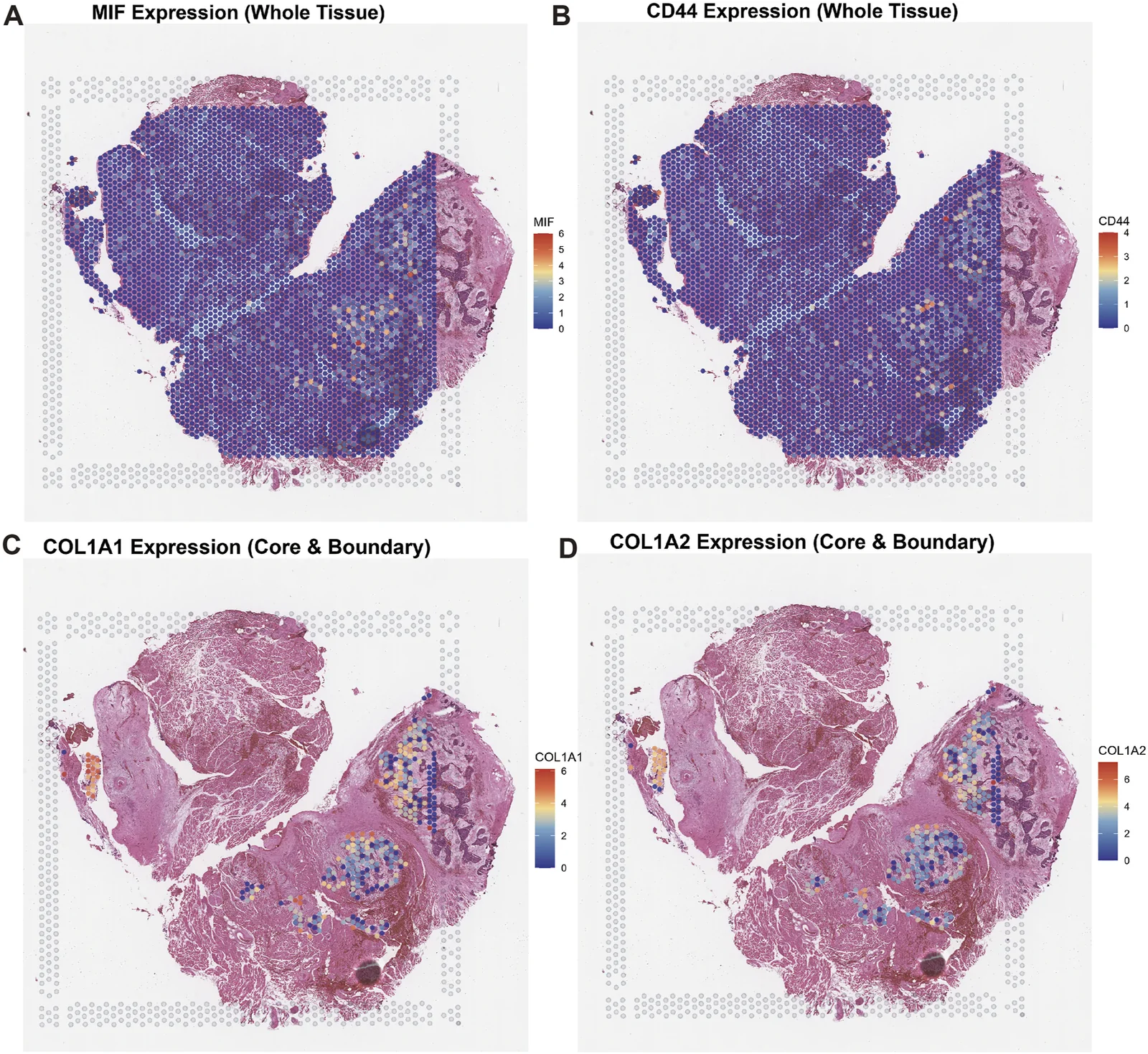
Spatial expression patterns of MIF, CD44, COL1A1, and COL1A2 in the analyzed LGACC section **(A,B)** Whole-tissue spatial expression maps of MIF and CD44, respectively. **(C,D)** COL1A1 and COL1A2 expression displayed within the manually annotated Tumor Core and Stroma Boundary regions.

To more directly illustrate the expression of collagen-related genes within the tumor-associated microenvironment, the spatial distributions of COL1A1 and COL1A2 were further visualized within the manually annotated Tumor Core and Stroma Boundary regions (Figures 5C,D). Within these two regions, both genes showed relatively higher expression in the Stroma Boundary. Because collagen transcripts may also be expressed in connective tissues outside the annotated tumor-associated regions, these spatial maps were intended to demonstrate the relative expression patterns within the selected tumor microenvironmental compartments rather than to establish that collagen expression was exclusively confined to the Stroma Boundary across the entire tissue section.

Collectively, these analyses provide hypothesis-generating evidence for candidate ECM-associated region-to-region interactions within the analyzed LGACC section. MIF-related signaling also emerged as an additional candidate program within the inferred region-to-region communication network. Nevertheless, whether the collagen-CD44 axis or MIF-related signaling contributes mechanistically to LGACC progression requires further validation through functional experiments.

### Bulk RNA-seq provides bulk-level context for spatially defined LGACC programs

To determine whether the spatially defined transcriptional programs identified in the Visium dataset were also reflected at the bulk transcriptomic level, we analyzed an independent publicly available bulk RNA-sequencing cohort comprising normal lacrimal gland tissues and LGACC samples (GSE228914). Differential expression analysis revealed marked transcriptomic differences between LGACC and normal tissues, with the majority of significantly differentially expressed genes showing increased expression in the tumor samples (Figure 1A).

We next examined representative genes associated with the spatially defined Tumor Core and Stroma Boundary programs. CD44 and CD74 showed trends toward increased expression in LGACC tissues, although neither difference reached statistical significance, whereas MIF showed no significant alteration at the bulk transcriptomic level (Figure 1B). In contrast, ECM-related genes, including COL1A1, COL6A1, and SPARC, exhibited heterogeneous expression across tumor samples and likewise did not reach statistical significance in this small cohort (Figure 1B). These findings suggest that bulk RNA sequencing may capture tumor-associated changes in receptor-related transcripts, whereas the localized ECM-associated program identified by spatial transcriptomics may not be fully preserved after tissue homogenization. To further investigate the relationship between bulk transcriptional alterations and the spatially defined programs, we performed gene set enrichment analysis using the signatures derived from the histopathology-guided spatial comparison. The Tumor Core-associated signature was positively enriched within the bulk transcriptional profile upregulated in LGACC, whereas the Stroma Boundary-associated signature showed negative enrichment relative to tumor tissues (Figure 1C). This pattern is compatible with the possibility that the Tumor Core transcriptional program is more readily captured in bulk tumor tissue, whereas the ECM-associated Stroma Boundary signal may be diluted by cellular-compositional heterogeneity within whole-tissue specimens.

Finally, ESTIMATE analysis showed greater overall variability in both immune and stromal scores among LGACC samples than among normal lacrimal gland tissues (Figure 1D). Given the limited cohort size and substantial intertumoral heterogeneity, these score-based comparisons were interpreted descriptively. Moreover, the small number of samples in this dataset, particularly the inclusion of only two normal controls, markedly limited the statistical power of the analysis. Collectively, the bulk RNA-sequencing results provide bulk-level context for the spatially defined Tumor Core-associated signature while also highlighting the limitations of bulk transcriptomic approaches in resolving the localized ECM-associated program of the Stroma Boundary.

### Proteomic analysis independently identifies enhanced ECM remodeling associated with LGACC-HGT

We next used an independently collected proteomic cohort to address a distinct biological question: whether high-grade transformation in LGACC is associated with alterations in ECM-related protein abundance. Whereas the spatial transcriptomic analysis compared histopathologically defined regions within a single tissue section, the proteomic analysis compared independent patient specimens with conventional LGACC and LGACC-HGT. This analysis was therefore designed to characterize protein-level alterations associated with high-grade transformation rather than to validate the spatial localization of the ECM-associated transcriptional pattern identified in the Visium section.

High-grade transformation (HGT), previously referred to as tumor dedifferentiation in adenoid cystic carcinoma, is characterized by the emergence of a high-grade malignant component that loses the typical morphological and functional features of conventional ACC and exhibits a less differentiated, more aggressive phenotype. HGT is therefore considered an important histopathological manifestation of malignant progression in ACC (Cheuk et al., 1999; Snyder et al., 1999).

Differential protein abundance analysis revealed a marked proteomic shift in LGACC-HGT, characterized by increased abundance of multiple ECM-related proteins (Figure 6A). Among the proteins enriched in LGACC-HGT, multiple collagen- and matrix-associated components were particularly prominent, including COL6A1, COL6A2, COL1A1, COL1A2, COL6A3, DCN, LUM, TNC, FMOD, ASPN, and MMP2 (Figures 6A,B).

**FIGURE 6:**
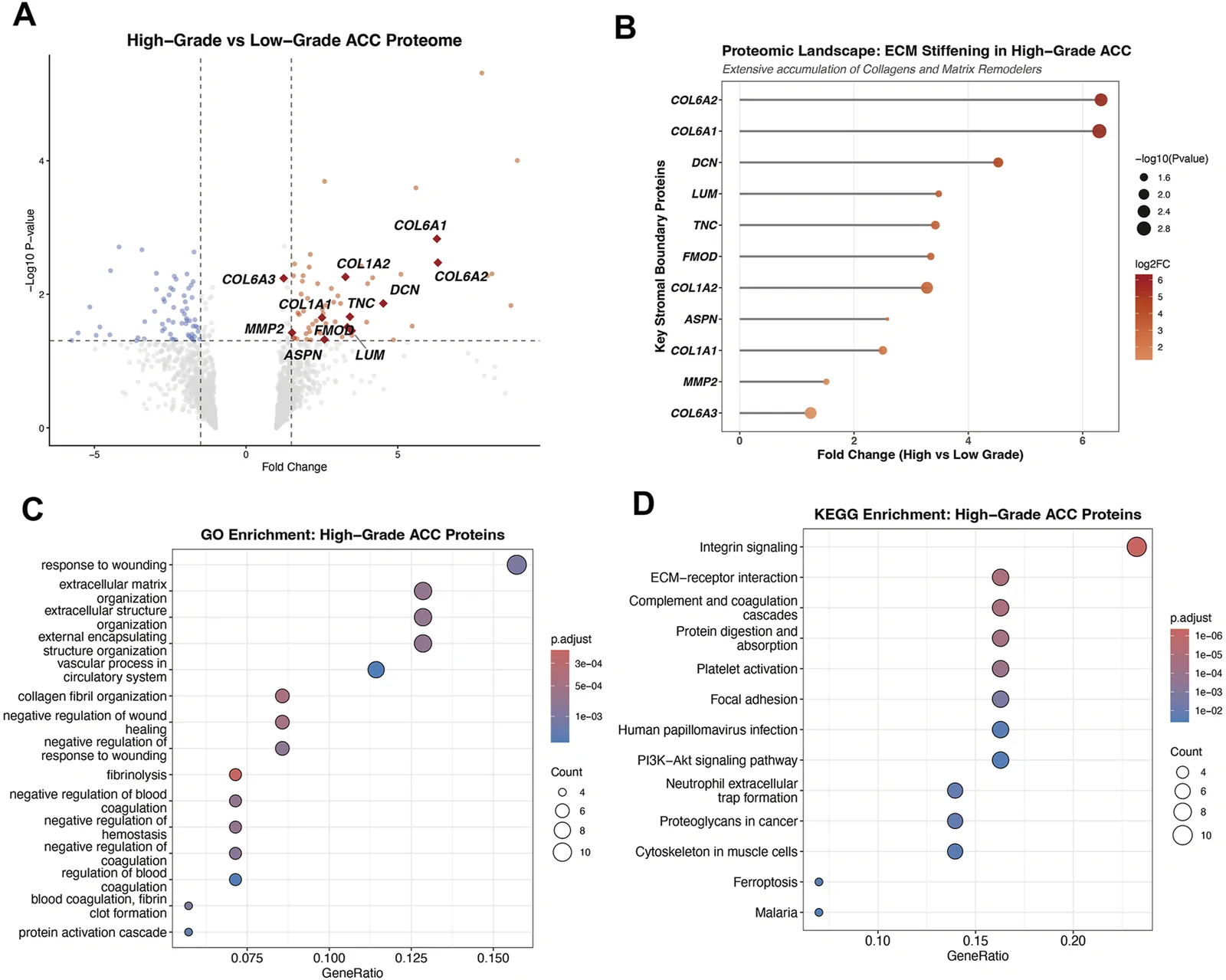
Proteomic profiling links LGACC-HGT to enhanced ECM-associated remodeling. **(A)** Volcano plot showing differentially abundant proteins between LGACC and LGACC-HGT samples. **(B)** Lollipop plot showing selected ECM-associated proteins increased in LGACC-HGT. **(C)** Gene Ontology enrichment analysis of proteins increased in LGACC-HGT. **(D)** KEGG pathway enrichment analysis of proteins increased in LGACC-HGT.

Functional enrichment analysis of proteins upregulated in LGACC-HGT further supported these findings. Gene Ontology enrichment analysis highlighted biological processes related to extracellular matrix organization, extracellular structure organization, external encapsulating structure organization, collagen fibril organization, and response to wounding (Figure 6C). KEGG pathway enrichment analysis further identified significant enrichment of integrin-related signaling, ECM-receptor interaction, focal adhesion, PI3K-Akt signaling, and proteoglycans in cancer (Figure 6D). Collectively, these findings indicate that high-grade transformation in this independently collected LGACC cohort is associated with enhanced ECM-related proteomic remodeling.

### Type I collagen coating promotes cell spreading and a migratory phenotype in ACC cells

To investigate whether the collagen-enriched microenvironment identified by spatial transcriptomic analysis directly influences ACC cell behavior, SACC-LM cells were cultured on plates coated with type I collagen at two surface densities (2 and 5 μg/cm^2^). Under standard culture conditions, untreated SACC-LM cells predominantly grew in compact clusters, with extensive cell-free areas between colonies. In contrast, cells cultured on collagen-coated surfaces exhibited a more uniformly spread morphology, accompanied by a marked reduction in the cell-free spaces between colonies (Figure 7A). Quantitative image analysis confirmed that the relative cell-free area was significantly smaller in both collagen-coated groups than in the control group, whereas no significant difference was observed between the two collagen coating densities (Figure 7B). CCK-8 assays showed no significant differences in cell viability among the control and collagen-coated groups at either 12 or 24 h (Figure 7C).

**FIGURE 7:**
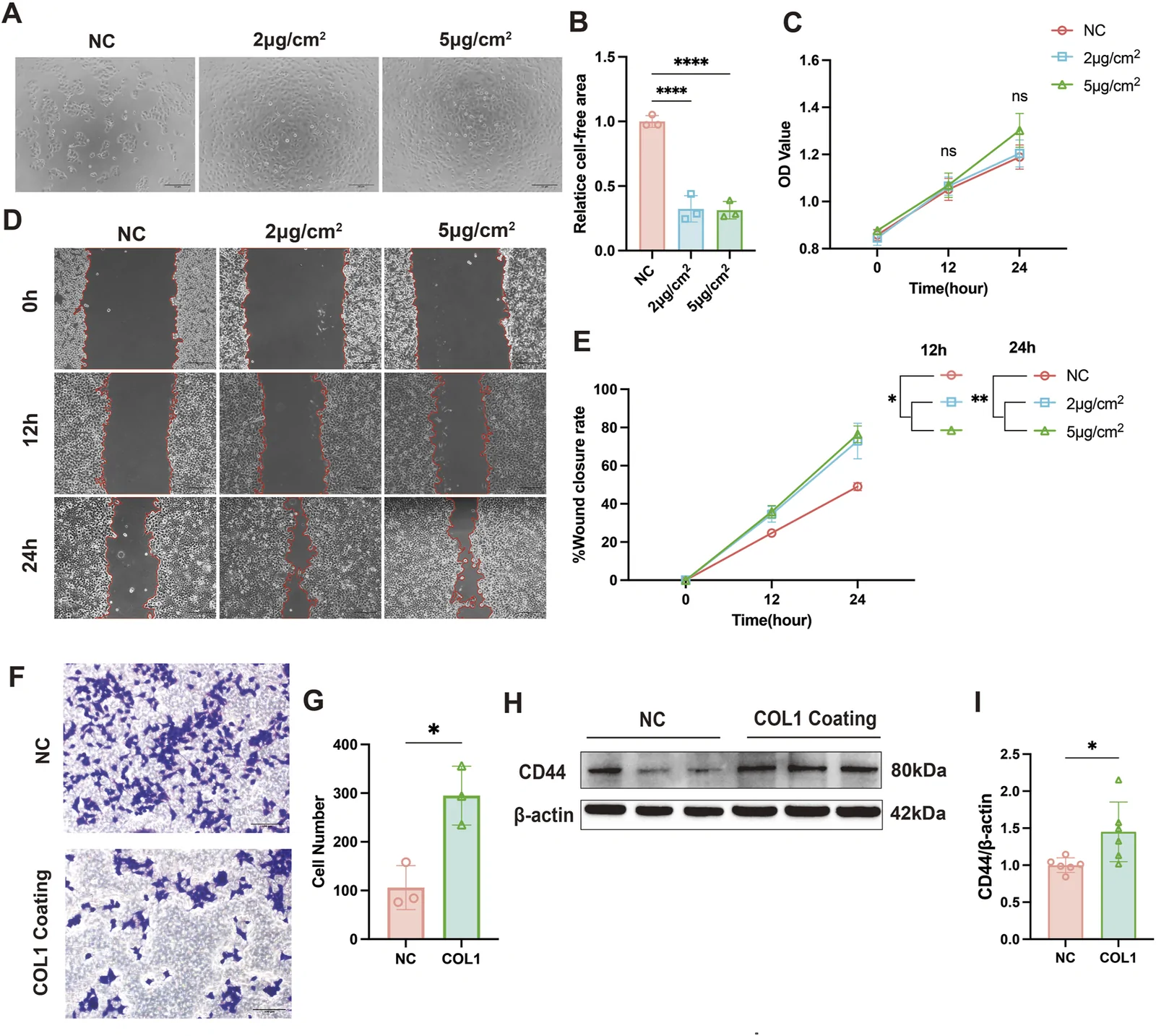
Type I collagen promotes SACC-LM cell spreading and migration. **(A)** Representative bright-field images showing the morphology of SACC-LM cells cultured on uncoated control plates (NC) or plates coated with type I collagen at 2 or 5 μg/cm^2^. **(B)** Quantification of the relative cell-free area from representative microscopic images. **(C)** CCK-8 assay showing cell viability at 0, 12, and 24 h. **(D)** Representative wound-healing images acquired at 0, 12, and 24 h after scratch generation. **(E)** Quantification of wound closure rates demonstrating enhanced migration in COL1-coated groups. **(F)** Representative images of migrated cells in the Transwell migration assay following crystal violet staining. **(G)** Quantification of migrated cell numbers showing significantly increased migration after COL1 coating. **(H)** Representative Western blot images showing CD44 protein expression in SACC-LM cells cultured on uncoated or COL1-coated plates. **(I)** Densitometric quantification of CD44 protein expression normalized to the loading control. *P < 0.05, **P < 0.01.

We next examined whether collagen exposure affected the migratory capacity of SACC-LM cells. Wound-healing assays showed that both collagen-coated groups exhibited more rapid wound closure than the untreated control group at 12 and 24 h (Figures 7D,E). Consistent with these findings, the Transwell assay demonstrated a significant increase in the number of cells that migrated through the membrane following collagen exposure (Figures 7F,G). Collectively, these results indicate that exposure to a collagen-rich extracellular matrix promotes a migratory phenotype in SACC-LM cells.

To assess whether the COL1-associated migratory response reflected nonspecific substrate adhesion, PLL was included as a non-collagen adhesive control. PLL did not alter wound closure compared with the uncoated control, whereas COL1 significantly increased wound closure. (Supplementary Figure S2).

Because the spatial communication analysis identified CD44 as a candidate receptor-associated signaling component within the collagen-enriched microenvironment, we further examined changes in CD44 protein expression following collagen exposure. Western blot analysis showed that CD44 protein expression was increased in collagen-treated cells compared with untreated control cells (Figures 7H,I). This finding provides preliminary experimental support for the spatial transcriptomic prediction that collagen-associated signaling may be accompanied by altered CD44 expression. However, the current data do not establish whether CD44 plays a direct mechanistic role in mediating collagen-induced cell migration.

### CD44 functional analysis

Because the spatial ligand-receptor analysis nominated CD44 as a candidate receptor-associated component of collagen-related regional interactions, we first examined whether CD44 protein expression was altered following COL1 exposure. Western blot analysis showed increased CD44 protein expression in cells cultured on type I collagen-coated surfaces compared with uncoated controls (Figures 7H,I).

To determine whether CD44 functionally contributes to the collagen-induced phenotype, we next performed siRNA-mediated CD44 knockdown in SACC-LM cells. Efficient reduction of CD44 protein expression was confirmed by Western blotting (Figure 8A). In control-transfected cells, COL1 coating increased migratory activity, consistent with the preceding experiments. In contrast, CD44 knockdown attenuated the COL1-induced increase in wound closure (Figures 8B,C). These findings indicate that CD44 contributes to the cellular response to a type I collagen-coated matrix, although they do not exclude the involvement of additional collagen receptors or downstream matrix-responsive pathways.

**FIGURE 8:**
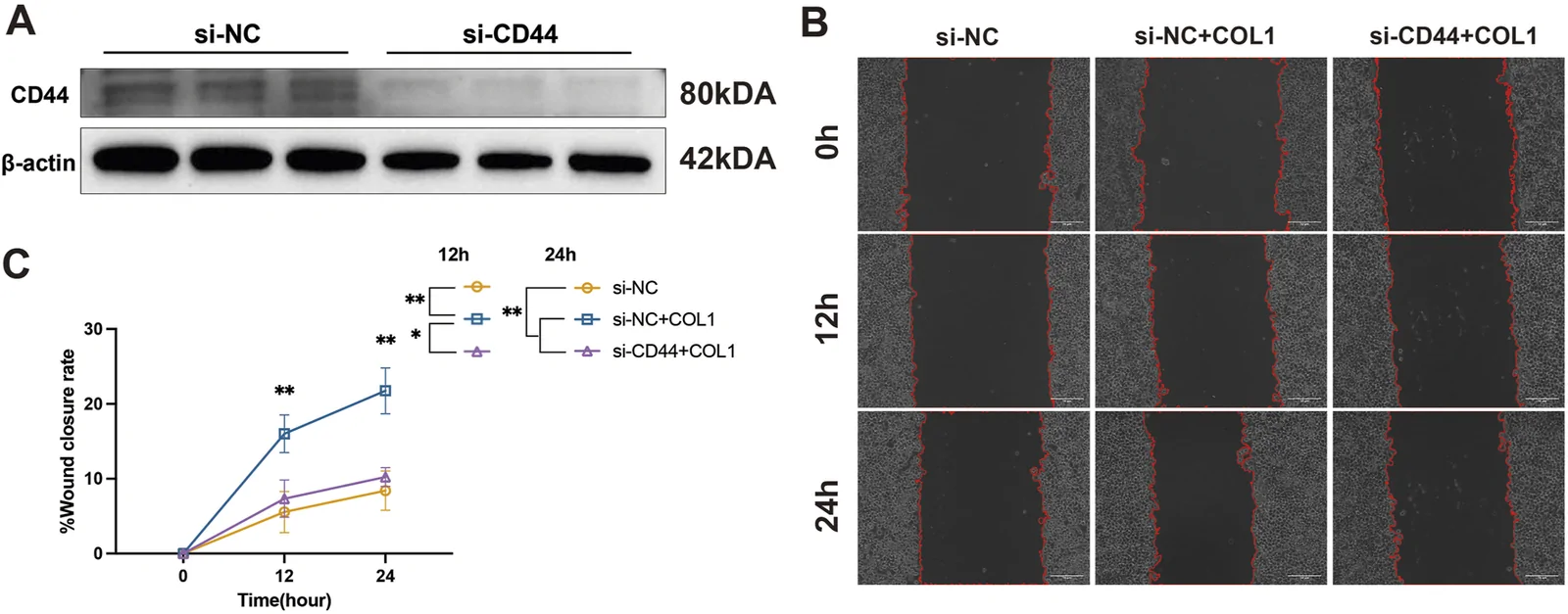
CD44 knockdown attenuates COL1-induced wound closure in SACC-LM cells. **(A)** Representative Western blot showing efficient siRNA-mediated knockdown of CD44 in SACC-LM cells. β-actin was used as the loading control. **(B)** Representative wound-healing images of cells transfected with negative-control siRNA (si-NC), si-NC cultured on type I collagen-coated surfaces (si-NC + COL1), and CD44-targeting siRNA cultured on type I collagen-coated surfaces (si-CD44 + COL1) at 0, 12, and 24 h. Red lines indicate the wound margins. **(C)** Quantification of wound closure at 12 and 24 h. COL1 coating significantly increased wound closure in si-NC-transfected cells, whereas CD44 knockdown attenuated the COL1-induced increase in wound closure. *P < 0.05, **P < 0.01.

## Discussion

LGACC is a rare epithelial malignancy characterized clinically by infiltrative local growth, a frequent propensity for perineural invasion, a high risk of local recurrence, and a marked lack of effective systemic treatment options (Emerick et al., 2022; Bradley and Bradley, 2013). Previous transcriptomic and proteomic studies have provided important insights into the molecular landscape of adenoid cystic carcinoma (Moeyersoms et al., 2023b; Yuan et al., 2026). However, conventional bulk-tissue analyses average signals derived from malignant cells, stromal cells, immune and vascular components, and the extracellular matrix, thereby obscuring the spatial context in which individual molecular programs operate. Spatial transcriptomics overcomes part of this limitation by enabling the investigation of regional transcriptional heterogeneity across intact tumor sections while preserving the underlying tissue architecture (Rao et al., 2021; Marx, 2021).

In the present study, we integrated histopathology-guided spatial transcriptomic analysis with publicly available bulk RNA-sequencing data, quantitative proteomic profiling of conventional LGACC and LGACC-HGT, and targeted *in vitro* functional experiments. Using this strategy, we identified a collagen- and ECM-associated transcriptional pattern within the histopathologically defined Stroma Boundary of the analyzed spatial transcriptomic section. Independently, proteomic analysis of conventional LGACC and LGACC-HGT demonstrated increased abundance of multiple ECM-related proteins in tumors with high-grade transformation. *In vitro* experiments further demonstrated that culture on a type I collagen-coated matrix altered the morphology and spatial organization of SACC-LM cells, promoted cell spreading and dispersal, and enhanced their migratory capacity. Notably, these phenotypic changes were not accompanied by a detectable increase in short-term cell viability. Taken together, these findings provide a hypothesis-generating spatial model in which ECM-associated stromal remodeling observed within the analyzed LGACC section may be biologically relevant to the collagen-responsive migratory phenotype observed in an ACC cell model.

The central spatial observation in the analyzed tissue section was an ECM-associated transcriptional pattern within the histopathologically defined Stroma Boundary adjacent to the highly cellular Tumor Core. Compared with the Tumor Core, the Stroma Boundary exhibited increased expression of collagen- and matrix-related genes, including COL1A1, COL1A2, SPARC, and DCN, together with enrichment of biological processes such as collagen fibril organization, extracellular matrix organization, and extracellular structure organization. Within the analyzed section, the tissue immediately adjacent to the tumor nests displayed a matrix-associated transcriptional state distinct from that of the highly cellular tumor region. In contrast, the Tumor Core showed relative enrichment of several tumor-associated genes, including MYC, CTNNB1, and MIF. However, the evidence supporting individual Tumor Core-associated genes was less consistent than that supporting the Stroma Boundary program. Although MIF and CTNNB1 showed directional trends toward enrichment in the initial regional comparison, these differences did not remain statistically significant after multiple-testing correction in the shared transcriptomic cluster restricted analysis. The Tumor Core-associated feature should therefore be interpreted more appropriately as a composite regional tendency rather than as definitive evidence that each individual candidate gene is consistently enriched.

In this study, matched H&E-stained images were used to guide spatial regional delineation, allowing the tissue to be explicitly partitioned into a highly cellular Tumor Core and a tumor-adjacent peritumoral stromal compartment, rather than relying exclusively on unsupervised graph-based clustering. Unsupervised clustering and histopathology-guided annotation capture two distinct aspects of spatial transcriptomic data. Graph-based clustering groups spots according to their global transcriptional similarity (Ståhl et al., 2016; Moncada et al., 2020), whereas morphology-guided annotation additionally incorporates tissue architecture, local cellularity, and the anatomical relationship between tumor nests and the surrounding stroma (Moncada et al., 2020; Lapuente-Santana et al., 2024).

This distinction is particularly important for the Visium platform. Each Visium spot captures a mixed transcriptional signal derived from multiple neighboring cells (Arora et al., 2023). In LGACC, tumor nests are often closely interwoven with collagen-rich stromal tissue. Consequently, unsupervised clustering alone may not precisely reconstruct histological boundaries. In the tissue section analyzed here, most spots from both the Tumor Core and Stroma Boundary were assigned to the shared transcriptomic cluster, and graph-based clustering did not sharply separate the two regions. However, this overlap does not diminish the relevance of the regional comparison. Rather, it suggests that these compartments may not represent transcriptionally discrete populations, but instead reflect distinct spatial transcriptional states embedded within a shared tumor-associated molecular background. Importantly, even when the analysis was restricted to the shared transcriptomic cluster, COL1A1, COL1A2, SPARC, and DCN remained significantly more highly expressed in Stroma Boundary spots than in Tumor Core spots, and the ECM-associated summary score was consistently higher in the Stroma Boundary. Thus, the observed ECM program cannot be explained simply by differences in graph-cluster composition. Accordingly, the Tumor Core and Stroma Boundary are more appropriately regarded as two morphologically defined spatial compartments than as independent molecular subtypes.

The principal spatial observation in the analyzed section was an ECM-associated transcriptional difference between the histopathologically defined Tumor Core and Stroma Boundary. Importantly, this comparison does not establish that the stromal ECM pattern is specifically induced by proximity to the tumor. The fibrous compartment surrounding the tumor nests was too spatially restricted to permit reliable subdivision into histologically comparable proximal and distal stromal zones at Visium resolution. Although connective-tissue regions were present farther from the tumor, their histological composition differed from that of the Stroma Boundary and therefore did not provide an appropriate distance-matched stromal control. Accordingly, the present findings should be interpreted as regional tumor-stromal transcriptional heterogeneity rather than as evidence of a proximity-dependent ECM program.

Independently of the spatial analysis, proteomic comparison between conventional LGACC and LGACC-HGT revealed increased abundance of multiple collagen- and matrix-associated proteins in LGACC-HGT, including COL1A1, COL1A2, COL6A1, COL6A2, COL6A3, DCN, LUM, TNC, FMOD, ASPN, and MMP2. Functional enrichment analysis of proteins upregulated in LGACC-HGT highlighted extracellular matrix organization, collagen fibril organization, ECM-receptor interaction, focal adhesion, integrin-related signaling, PI3K-Akt signaling, and proteoglycan-associated pathways. These findings indicate that high-grade transformation in the proteomic cohort was accompanied by enhanced ECM-related remodeling.

Importantly, the proteomic and spatial analyses address different biological comparisons. The proteomic dataset lacks spatial resolution and therefore cannot determine whether the differentially abundant ECM proteins originate specifically from the histopathologically defined Stroma Boundary, nor can it establish the reproducibility of the spatial pattern across patients. Rather, the two datasets independently identify ECM-related alterations in distinct analytical contexts: regional transcriptional heterogeneity within the analyzed spatial section and proteomic differences associated with high-grade transformation across patient specimens. Similarly, although ECM-receptor interaction, focal adhesion, integrin-related signaling, and PI3K-Akt signaling were enriched in the LGACC-HGT proteomic dataset, these findings arose from an independent inter-sample comparison and should not be interpreted as evidence that these pathways mediate the response to COL1 in SACC-LM cells. Whether collagen exposure activates integrin-FAK or related matrix-responsive signaling pathways therefore remains to be determined experimentally. Given the increased abundance of COL6A1 and COL6A2 in LGACC-HGT, the effects of collagen VI and other ECM components on ACC cell behavior also warrant further investigation. Together, these observations motivate further investigation of ECM remodeling and matrix-responsive signaling in LGACC without implying direct cross-platform validation or a defined downstream mechanism.

The ECM is increasingly recognized as an active regulator of tumor behavior rather than merely a passive consequence of tumor growth (Winkler et al., 2020; Mariathasan et al., 2018). In solid tumors, collagens, proteoglycans, matricellular proteins, and matrix-remodeling enzymes can influence cell adhesion, migration, proliferation, survival, mechanotransduction, growth factor availability, vascular function, and immune organization (Pickup et al., 2014; Kai et al., 2019). In breast tumor models, progressive reorganization of collagen fibers at the tumor-stromal interface can generate radially aligned fiber bundles that provide permissive tracks for local tumor cell invasion (Provenzano et al., 2006). Subsequent studies further demonstrated that increased stromal collagen density accelerates mammary tumor development and metastasis, whereas enhanced collagen crosslinking increases matrix stiffness and promotes integrin-dependent focal adhesion signaling and malignant progression (Provenzano et al., 2008; Levental et al., 2009). In human breast cancer, collagen deposition, fiber linearization, and stromal stiffening have all been associated with more aggressive tumor phenotypes, while collagen fibers aligned perpendicular to the tumor boundary have been linked to poorer patient survival (Acerbi et al., 2015; Conklin et al., 2011). In desmoplastic tumors, excessive matrix deposition may alter tissue stiffness and architecture, facilitate directional migration, remodel tumor-stromal interactions, and affect the penetration and therapeutic efficacy of anticancer agents (Stylianopoulos et al., 2018; Jain, 2013; Huang et al., 2021; Wolf et al., 2020). In pancreatic cancer models, type I collagen has been shown to promote polarized cell morphology, adhesion, motility, and Transwell migration through integrin α2β1-dependent focal adhesion kinase signaling (Lu et al., 2014). Other studies have reported that type I collagen can induce epithelial–mesenchymal transition and enhance pancreatic cancer cell migration and invasion through crosstalk between β1-integrin signaling and the Hedgehog pathway (Duan et al., 2014). These observations are consistent with the morphological redistribution and enhanced migratory capacity of SACC-LM observed under type I collagen-coated conditions in the present study.

The *in vitro* experiments provided functional support for this interpretation. Under uncoated culture conditions, SACC-LM cells predominantly formed compact aggregates separated by extensive cell-free areas. In contrast, when cultured on type I collagen-coated matrices, the cells adopted a markedly more dispersed and spread morphology, accompanied by a significant reduction in the cell-free area. CCK-8 analysis revealed no significant differences in cell viability among the experimental groups at either 12 or 24 h. This finding indicates that the reduction in cell-free area was unlikely to result from a short-term increase in cell proliferation or cell number. Instead, it more likely reflected altered cell-matrix adhesion, enhanced cell spreading, and redistribution of cells across the culture surface. In other words, COL1 exposure altered the way the cells occupied the matrix surface rather than increasing the number of cells. Type I collagen coating also enhanced the migratory capacity of SACC-LM cells. In the wound-healing assay, both collagen-coated groups exhibited greater wound closure than the uncoated control group at 12 and 24 h. The Transwell assay provided complementary evidence, showing a significantly greater number of cells traversing the membrane under COL1-coated conditions than under control conditions. When considered together with the absence of detectable changes in the short-term CCK-8 signal, these complementary functional assays indicate that a collagen-rich matrix promotes a migratory phenotype in SACC-LM. Taken together, these independent observations across distinct analytical contexts motivate further investigation of stromal collagen remodeling as a candidate feature of LGACC biology, while its reproducibility across patients remains to be established.

The spatial communication analysis further nominated CD44 as a candidate receptor-associated component of collagen-related regional interactions. The inferred signaling network indicated that multiple collagen- and matrix-related ligands were predicted to originate from the Stroma Boundary and signal toward the Tumor Core, with CD44 positioned on the receiving side of these interactions. CD44 is a multifunctional cell-surface adhesion receptor that mediates cell-matrix interactions in various malignancies and has been implicated in the regulation of migration, invasion, survival, and phenotypic plasticity (Wolf et al., 2020; Ponta et al., 2003; Chen et al., 2018.; Misra et al., 2011). Consistent with the spatial prediction, COL1 exposure was accompanied by increased CD44 protein expression in the *in vitro* model. More importantly, CD44 knockdown attenuated the collagen-induced migratory phenotype, providing functional evidence that CD44 contributes to the cellular response to the collagen-coated matrix. This concordance between computational inference and experimentally measured protein expression provides biological plausibility for the hypothesis that CD44 may participate in the cellular response to a collagen-enriched microenvironment. Nevertheless, this effect should not be interpreted as establishing CD44 as the exclusive collagen-responsive receptor, as collagen-integrin interactions and other matrix-dependent signaling mechanisms may operate in parallel.

MIF-related signaling also emerged from the spatial analysis as a candidate pathway. Previous studies have implicated MIF in tumor cell survival, inflammatory signaling, immune regulation, proliferation, and resistance to apoptosis across multiple malignancies (Bucala and Donnelly, 2007; Balogh et al., 2018). In the present dataset, although MIF showed relative enrichment in the Tumor Core in the initial regional comparison, this difference was no longer statistically significant after multiple-testing correction when the analysis was restricted to the shared transcriptomic cluster. Moreover, its spatial expression was heterogeneous and was not strictly confined to the Tumor Core. Therefore, in the absence of robust functional validation, MIF should be regarded as a tumor-associated candidate molecule that warrants further investigation rather than as an established driver of LGACC. Whether MIF-related signaling is directly linked to the collagen-associated ECM program also remains unresolved and will require clarification in future studies.

The publicly available bulk RNA-sequencing dataset provided additional contextual support for the spatial findings while also illustrating the inherent limitations of tissue homogenization-based approaches. Within this bulk transcriptomic cohort, several candidate genes exhibited substantial intertumoral variability, and not all ECM-related genes enriched at the spatial level reached statistical significance when LGACC samples were compared with normal lacrimal gland tissues. Gene set enrichment analysis further indicated that the Tumor Core-associated program was more readily detectable in bulk tumor specimens, whereas the Stroma Boundary-associated signature was not consistently captured. This pattern is biologically plausible. In homogenized tissue samples, transcriptional signals contributed by densely packed tumor cells are likely to predominate, whereas localized stromal signals may be diluted by variations in tumor purity, stromal content, and sampling location. Nevertheless, this bulk RNA-sequencing cohort included only five LGACC samples and two normal lacrimal gland controls, and the potential influence of intersample differences in tissue composition cannot be overlooked. Accordingly, these data should be regarded as a cross-platform reference rather than as direct validation of the spatially defined compartments. More broadly, concordance among spatial transcriptomics, bulk RNA sequencing, and proteomics is most appropriately interpreted at the level of biological programs and pathways, rather than by expecting a one-to-one correspondence for every regionally expressed gene.

In summary, histopathology-guided analysis of a single spatially profiled LGACC section identified an ECM-associated transcriptional pattern within the analyzed LGACC section. This within-section pattern was characterized by relatively higher expression of collagen- and matrix-related genes and remained detectable when the analysis was restricted to the dominant graph-based cluster shared by the two annotated regions. Separately, proteomic analysis showed that LGACC-HGT was associated with enhanced ECM-related protein remodeling. Functional experiments further showed that exposure to type I collagen altered the organization of SACC-LM cells, promoted cell spreading and dispersal, and enhanced their migratory capacity without significantly altering short-term cell viability. Spatial communication analysis, together with protein-level measurements, additionally identified CD44 as a biologically plausible candidate associated with the cellular response to a collagen-rich environment, although its mechanistic role remains to be established. Collectively, these findings provide a hypothesis-generating, spatially informed framework for investigating whether ECM-associated stromal remodeling contributes to the migratory and locally aggressive behavior of LGACC.

These observations may also have potential translational implications, although the evidence remains preliminary. Therapeutic strategies for adenoid cystic carcinoma have historically focused predominantly on molecular alterations intrinsic to tumor cells (Powell et al., 2023; Li and Ma, 2025), whereas the contribution of stromal components has received comparatively limited attention. The identification of an ECM-rich compartment immediately adjacent to the tumor, together with the association between enhanced ECM remodeling and high-grade transformation, suggests that the tumor microenvironment may participate in shaping the locally invasive behavior of LGACC. Moreover, the finding that exposure to type I collagen alone was sufficient to alter tumor cell organization and enhance migration further supports the functional relevance of the matrix environment. Based on the observations in this study, potential intervention strategies may be considered at several levels, including modulation of excessive ECM deposition or remodeling, disruption of tumor cell–matrix recognition through receptors such as CD44 or integrins, and inhibition of downstream matrix-responsive signaling pathways, including focal adhesion-associated and PI3K-Akt-related signaling.

However, the clinical feasibility of these approaches requires careful consideration. ECM components and their receptors are widely involved in normal tissue architecture and homeostasis, raising the possibility of on-target toxicity with systemic inhibition. Moreover, the present study does not establish that CD44, integrins, or any specific downstream signaling pathway is required for LGACC progression. Therefore, these candidate targets should currently be regarded as directions for further investigation rather than immediately actionable therapeutic targets. Given the predominantly local infiltrative behavior of LGACC, matrix-directed strategies may ultimately be more suitable as adjunctive approaches aimed at limiting local invasion or recurrence in combination with surgery or radiotherapy, rather than as stand-alone therapies. Interventions directed against the matrix, matrix receptors, or matrix-dependent signaling pathways will require validation in more physiologically relevant experimental systems, including three-dimensional culture models, patient-derived organoids, co-culture systems, and *in vivo* models.

Several limitations should be considered when interpreting these findings. First, the spatial transcriptomic analysis was based on a single publicly available LGACC tissue section. The individual spots therefore represented spatial observational units within one specimen rather than independent biological replicates. Spot-level P values quantify regional separation within this section but do not estimate interpatient variability or establish that the identified pattern is reproducible across LGACC cases. Accordingly, the spatial findings should be regarded as hypothesis-generating, and validation in independent spatial cohorts is required. Second, the cellular sources of the ECM-related transcripts could not be resolved because each Visium spot captures mixed transcriptional signals from multiple neighboring cells. The limited spatial extent and resolution of the available tissue section did not permit reliable subdivision of the fibrous stromal compartment into histologically comparable tumor-adjacent and distal regions. Consequently, the present dataset cannot determine whether ECM-associated transcriptional differences vary as a function of distance from the tumor. Future studies using larger tissue sections and higher-resolution spatial technologies will be required to directly test this question. Single-cell sequencing, higher-resolution spatial technologies, or spatial proteomics will be required to identify the cell populations responsible for these signals. Third, the SACC-LM cell line used in the functional experiments was not derived from lacrimal gland ACC. Differences in molecular characteristics and biological behavior between this cell line and primary LGACC tissues may therefore limit the direct generalizability of the *in vitro* findings. Fourth, the collagen-coating model represents a simplified two-dimensional system that cannot reproduce the three-dimensional architecture, matrix mechanics, cellular complexity, or immune microenvironment of the native tumor. Although the wound-healing and Transwell assays consistently supported enhanced motility, they cannot fully distinguish the respective contributions of altered adhesion, cell spreading, directional migration, and invasion. Finally, although CD44 knockdown attenuated the collagen-responsive phenotype, the absence of rescue experiments precludes definitive attribution of this response specifically to CD44. Similarly, MIF-related signaling remains a spatially inferred candidate pathway without functional validation.

Taken together, this study integrates a within-section spatial observation with independent proteomic and *in vitro* findings to highlight ECM-associated stromal remodeling as a potentially relevant feature for further investigation in LGACC. The spatial analysis identified a collagen-enriched Stroma Boundary adjacent to the Tumor Core within the analyzed section, while the independent proteomic and functional datasets highlighted ECM remodeling and collagen-responsive cell behavior in distinct experimental contexts. The observed effects of type I collagen on tumor cell organization, spreading, and migration further support the functional relevance of this matrix-rich compartment, while the identification of CD44 provides a biologically plausible candidate through which SACC-LM cells may respond to collagen-associated cues. Overall, the present study identifies ECM-associated transcriptional heterogeneity between histopathologically defined tumor and stromal compartments within an LGACC section and places this observation alongside independent proteomic and functional evidence implicating ECM-related biology in LGACC. These findings provide a spatially informed framework for further investigating tumor–matrix interactions while highlighting the need for future spatial datasets capable of directly resolving distance-dependent stromal remodeling.

## Data Availability

The datasets presented in this study are deposited in the NCBI Gene Expression Omnibus (GEO) repository under accession numbers GSE228685 and GSE228914. The spot-level histopathology-guided annotation metadata and R code used for the regional spatial analysis are deposited in the Zenodo repository, DOI: 10.5281/zenodo.22113882.

## Supplementary material

SUPPLEMENTARY FIGURE S1 Cluster 5-restricted analysis supports an ECM-associated transcriptional program in tumor-proximal stroma. **(A)** Spatial distribution of graph-based clusters generated in the original Loupe Browser analysis. **(B)** Relative composition of graph-based clusters across histopathology-guided tumor core and tumor-proximal stromal regions. Cluster 5 represented the dominant shared cluster in both regions. **(C)** Expression of representative ECM-associated genes after restricting analysis to spots assigned to Cluster 5. **(D)** ECM-associated and core-associated module scores in tumor core and tumor-proximal stroma within Cluster 5.

SUPPLEMENTARY FIGURE S2 Comparison of type I collagen and poly-L-lysine effects on SACC-LM cell migration and viability. **(A)** Representative wound-healing images of SACC-LM cells cultured under uncoated control, type I collagen (COL1)-coated, and poly-L-lysine (PLL)-coated conditions. **(B)** Quantification of wound closure showing no significant difference between the uncoated and PLL-coated groups, whereas COL1 significantly increased wound closure compared with both groups.
