# Supplementary material for: Histopathology-guided spatial transcriptomic analysis identifies an ECM-associated stromal pattern in lacrimal gland adenoid cystic carcinoma with complementary proteomic and functional analyses
Source: Front Pharmacol. 2026 Oct 9;17:1949721. doi: 10.3389/fphar.2026.1949721 (PMC13690537; doi:10.3389/fphar.2026.1949721)
Supplement: Supplementary file 1 [file Table1.docx]

| ID | Sex | Age (years) | Laterality | Disease duration (months) | Imaging findings | TNM stage | LGACC-HGT | Treatment | Follow-up (months) | Metastasis | Survival status |
| --- | --- | --- | --- | --- | --- | --- | --- | --- | --- | --- | --- |
| 1 | Female | 68 | OD | 24 | Mass in the superolateral right orbit with invasion of the extraocular muscles | T2N0M0 | No | Surgery | Lost to follow-up | NA | NA |
| 2 | Female | 29 | OS | 12 | Left intraorbital mass | T2N0M0 | No | Surgery + radiotherapy | 43 | None | Deceased |
| 3 | Male | 49 | OD | 12 | Right intraorbital mass with orbital bone involvement and bone destruction, invading intracranial structures and extraocular muscles | T4aN0M0 | No | Surgery+ radiotherapy + chemotherapy | 180 | None | Alive |
| 4 | Male | 65 | OS | 6 | Mass in the superolateral left orbit | T2N0M0 | No | Surgery + radiotherapy | 22 | None | Alive |
| 5 | Male | 43 | OD | 6 | Mass in the superolateral right orbit with periosteal involvement and bone destruction, invading the temporal fossa, paranasal sinuses, and extraocular muscles | T4cN0M0 | Yes | Surgery + radiotherapy | 96 | Lung metastasis | Deceased |
| 6 | Female | 50 | OS | 6 | Left intraorbital mass with periosteal involvement and bone destruction, with intracranial invasion | T4cN0M0 | Yes | Surgery + radiotherapy | 17 | Bone metastasis | Deceased |
| 7 | Female | 35 | OD | 2 | Right intraorbital mass | T3N0M0 | Yes | Surgery + radiotherapy | 54 | None | Alive |
| 8 | Male | 49 | OD | 2 | Mass in the superolateral right orbit invading the lateral rectus, superior rectus, and lateral orbital wall | T4cN0M0 | Yes | Surgery | 5 | None | Deceased |

**Supplementary Table 1.** Clinicopathological characteristics of patients included in the quantitative proteomic cohort.

Note: LGACC-HGT, lacrimal gland adenoid cystic carcinoma with high-grade transformation; TNM, tumor–node–metastasis; T, primary tumor; N, regional lymph node; M, distant metastasis; NA, not available.
